# Rapid SARS-CoV-2 Intra-Host and Within-Household Emergence of Novel Haplotypes

**DOI:** 10.3390/v14020399

**Published:** 2022-02-15

**Authors:** Laura Manuto, Marco Grazioli, Andrea Spitaleri, Paolo Fontana, Luca Bianco, Luigi Bertolotti, Martina Bado, Giorgia Mazzotti, Federico Bianca, Francesco Onelia, Giovanni Lorenzin, Fabio Simeoni, Dejan Lazarevic, Elisa Franchin, Claudia Del Vecchio, Ilaria Dorigatti, Giovanni Tonon, Daniela Maria Cirillo, Enrico Lavezzo, Andrea Crisanti, Stefano Toppo

**Affiliations:** 1 Department of Molecular Medicine, University of Padova, 35121 Padua, Italy; laura.manuto@unipd.it (L.M.); marco.grazioli@unipd.it (M.G.); martina.bado@studenti.unipd.it (M.B.); giorgia.mazzotti@studenti.unipd.it (G.M.); federico.bianca@phd.unipd.it (F.B.); francesco.onelia@studenti.unipd.it (F.O.); elisa.franchin@unipd.it (E.F.); claudia.delvecchio@unipd.it (C.D.V.); enrico.lavezzo@unipd.it (E.L.); 2 Center for Omics Sciences, IRCCS San Raffaele Institute, 20132 Milan, Italy; spitaleri.andrea@hsr.it (A.S.); simeoni.fabio@hsr.it (F.S.); lazarevic.dejan@hsr.it (D.L.); tonon.giovanni@hsr.it (G.T.); 3 Research and Innovation Center, Edmund Mach Foundation, 38098 San Michele all’Adige, Italy; paolo.fontana@fmach.it (P.F.); luca.bianco@fmach.it (L.B.); 4 Department of Veterinary Sciences, University of Torino, Grugliasco, 10095 Turin, Italy; luigi.bertolotti@unito.it; 5 Emerging Bacterial Pathogens Unit, Division of Immunology, Transplantation and Infectious Diseases, IRCCS San Raffaele Scientific Institute, 20132 Milan, Italy; lorenzin.giovanni@hsr.it (G.L.); cirillo.daniela@hsr.it (D.M.C.); 6 Microbiology and Virology Diagnostic Unit, Padua University Hospital, Via Giustiniani, 35121 Padova, Italy; 7 MRC Centre for Global Infectious Disease Analysis, Imperial College London, London SW7 2BX, UK; i.dorigatti@imperial.ac.uk; 8 Functional Genomics of Cancer Unit, Division of Experimental Oncology, IRCCS San Raffaele Scientific Institute, 20132 Milan, Italy; 9 Department of Life Sciences, Imperial College London, London SW7 2BX, UK; 10 CRIBI Biotech Center, University of Padova, 35121 Padova, Italy

**Keywords:** SARS-CoV-2, epidemiology, viral genomics, intra-host haplotypes, longitudinal analysis, NGS sequencing, minimum spanning network, phylogenetic analysis

## Abstract

In February 2020, the municipality of Vo’, a small town near Padua (Italy) was quarantined due to the first coronavirus disease 19 (COVID-19)-related death detected in Italy. To investigate the viral prevalence and clinical features, the entire population was swab tested in two sequential surveys. Here we report the analysis of 87 viral genomes, which revealed that the unique ancestor haplotype introduced in Vo’ belongs to lineage B, carrying the mutations G11083T and G26144T. The viral sequences allowed us to investigate the viral evolution while being transmitted within and across households and the effectiveness of the non-pharmaceutical interventions implemented in Vo’. We report, for the first time, evidence that novel viral haplotypes can naturally arise intra-host within an interval as short as two weeks, in approximately 30% of the infected individuals, regardless of symptom severity or immune system deficiencies. Moreover, both phylogenetic and minimum spanning network analyses converge on the hypothesis that the viral sequences evolved from a unique common ancestor haplotype that was carried by an index case. The lockdown extinguished both the viral spread and the emergence of new variants.

## 1. Introduction

Severe acute respiratory syndrome coronavirus 2 (SARS-CoV-2) infection continues to spread world-wide, with over 5.7 million deaths out of the more than 386 million positive cases reported since the beginning of the pandemic [1]. There is extensive evidence that the SARS-CoV-2 genome has evolved and has acquired several mutations conferring higher viral fitness, with almost all genomic sites being affected by mutation events [2,3,4]. Examples of viral evolution are the different variants of concern that emerged since the beginning of the pandemic, such as the Alpha variant (originally identified in the UK), the Beta variant (originally identified in South Africa), the Gamma variant (originally identified in Brazil), the Delta variant (originally identified in India), and the Omicron variant (originally identified in South Africa). The first mutation known to improve viral fitness was Spike D614G [5], which was first identified in the Alpha variant and then reached a prevalence of nearly 100% globally. This mutation was correlated to higher viral replication, as the mutation N501Y [6], which was also associated with a higher affinity for the receptor ACE2 and was identified in the Alpha, Beta, and Gamma variants. Another mutation of note is E484K, which is found in the Beta and Gamma variants of concern and is related to a decrease in the susceptibility to monoclonal antibodies and convalescent plasma [7]. Overall, the number of mutations defining a lineage has gradually increased, with the Delta variant being defined by over 30 mutations and Omicron showing over 70 mutations, most of which are non-synonymous. The mutation patterns defining the different variants confer specific advantages. Some variants showed higher pathogenicity (Alpha, Gamma, and Delta), while others improved the immune escape capability and promoted reinfections (Beta and Omicron) or increased transmissibility (Alpha, Beta, Gamma, Delta, and Omicron) [8,9].

Here we provide novel insights into the generation of diversity of SARS-CoV-2 genomes in a close community.

In February and March 2020, preceding and following a two week lockdown, respectively, two mass swab testing campaigns were conducted in Vo’ to trace and isolate all the positive subjects. At the surveys, metadata regarding symptom onset and contacts were also collected. Based on these data, we previously investigated the effectiveness of the implemented non-pharmaceutical interventions (NPIs), the role of asymptomatic infections [10], and the tracking of T-cell signatures of the whole population [11]. In May and November 2020, two follow-ups were carried out on the same population to assess the antibody dynamics following infections [12].

Here, we report on the results from the sequencing of the SARS-CoV-2 genomes extracted from the oro-nasopharyngeal swabs of positive subjects. We obtained the sequences for 87 samples, representing the vast majority of the positive subjects detected in Vo’. The sequencing data has allowed us to unequivocally identify the ancestor haplotype of the virus circulating in Vo’ at the time of the outbreak. This evidence provided the opportunity to infer the evolution of SARS-CoV-2 in an isolated community, from an intra-host, a household, and a general perspective, tracing the generation of novel mutations from the index haplotype. Information on the date of symptom onset allowed us to reconstruct a temporal expanding network of infected subjects. The uniqueness of this study lies in the feature of this isolated and small community that, since the very beginning of the SARS-CoV-2 pandemic, was monitored over time, enabling us to investigate the virus evolution and transmission dynamics.

## 2. Materials and Methods

### 2.1. Sample Collection, Library Construction, and Sequencing Methods

Total nucleic acids were purified from nasopharyngeal swab samples using a MagNA Pure 96 System (Roche Applied Sciences). All samples were treated with DNase I (Promega, Madison, WI, USA) to eliminate the residues of the host and bacterial gDNA. Quantity and quality checks were performed on the HSRNA chip of a Tapestation 4100 (Agilent, Santa Clara, CA, USA). The samples with low RNA concentrations were concentrated using a Vacufuge plus (Eppendorf, Hamburg, Germany). Starting with 50 ng of total RNA, NEBNext Ultra II First Strand and Non-Directional RNA Second Strand Synthesis Module (NEB, Ipswich, Massachusetts) were used to convert RNA into cDNA. Before proceeding with the next steps, we performed RTqPCR on all samples to determine the relative abundance of viral RNA. RTqPCR was performed by amplification regions for the following target genes: RNA-dependent RNA-polymerase (RdRP), nucleocapsid (N), envelope (E), and the internal control.

The enrichment step was performed according to the manufacturer’s instructions. We used the Illumina Nextera Flex kit for Enrichment/Respiratory Virus Oligos Panel Detection of SARS-CoV-2 RNA. Briefly, the samples were fragmented and barcoded with Nextera Flex, followed by PCR amplification (17 cycles). Before hybridizations with the Respiratory Virus Oligos Panel, the samples were pooled according to the viral load (previously determined by RTqPCR). Each pool was composed of eight samples with similar Ct (threshold cycle) to avoid bias competition between samples with low and high Ct during hybridization and sequencing.

After a quality check on the HSDNA chip of a Tapestation 4100(Agilent), the libraries were diluted to 2.2pM, mixed with Pfix (20%), and then loaded on a NextSeq 500 sequencer (Illumina, San Diego, CA, USA). Sequencing was performed in PE (pair-end) mode, generating 149nt length reads and 1-5M clusters for each sample.

### 2.2. Viral Genome Assembly: Quality Check and Mapping of the Reads

The raw sequences were filtered for length and quality with Trimmomatic v0.40 according to the following parameters: ILLUMINACLIP:TruSeq3-PE-2:2:30:10, LEADING:30, TRAILING:30, SLIDINGWINDOW:4:20, and MINLEN:90. High quality reads were aligned on the SARS-CoV-2 reference genome (genbank ACC: NC_045512) with BWA-MEM v0.7.1. Duplicated reads were then removed with Picard tool v2.25.0 (http://broadinstitute.github.io/picard/, accessed on 17 January 2022). Consensus sequences were generated using a combination of SAMtools v1.11 and VarScan v2.4.1 variant caller. The consensus sequences were reconstructed from the VarScan output with an in-house script that automatically introduces ‘N’ in low quality or uncertain/uncovered regions of the reference sequence. The 87 SARS-CoV-2 sequences produced in this study were submitted to the GISAID portal (www.gisaid.org on 24 February 2021). Table S2 reports the correspondence of the GISAID IDs of the newly produced sequences with the identifiers reported in this paper.

### 2.3. Sequence Selection from GISAID

The Vo’ genome sequences of SARS-CoV-2, divided by lineage, were used as the input of cd hit-est-2d [13] and were compared with the GISAID database. The first 20 most similar genomes of every cluster were used as the input of MAFFT [14] to produce a multiple alignment with the Wuhan genome (GenBank ID MN908947.3) as a reference. This dataset has been used to build the minimum spanning network (MSN) and the phylogenetic tree. Table S2 reports the GISAID IDs of the sequences utilized in this paper.

### 2.4. Minimum Spanning Network (MSN)

The MSN [15] was built to find out the most probable path of contagion, starting from a distance matrix calculated by the genome sequences of lineage B, as classified by the Pangolin tool (https://github.com/cov-lineages/pangolin, accessed on 8 July 2021). The nodes of the MSN represent the genomes that share the same pattern of SNPs (with reference to the Wuhan genome MN908947.3), while an edge connects two nodes if they have compatible genomes (e.g., one can be obtained from the other by adding some mutations). Nodes are labeled with the sampling location and date of the corresponding sequences and edges are labeled with the number of mutations that differentiate the two sets of connected sequences. The minimum spanning network algorithm used to reconstruct the MSN from the distance matrix connects the virus sequences, minimizing the edit distance (Hamming distance). In the reconstructed network, we defined a possible introduction as a node also containing sequences sampled outside the Vo’ area. Additionally, the sampling date of the Vo’ node (i.e., the introduction) must be subsequent to that of the other GISAID sequences. Similarly, a possible exit is a node containing only Vo’ sequences that are the source of an edge leading to a node containing only GISAID sequences (i.e., and sequence not from Vo’). Moreover, the sampling date of the Vo’ node must precede that of the GISAID sequences. The MSNs were finally pruned to remove nodes not directly connected to a node containing sequences from Vo’.

### 2.5. Phylogenetic Analysis

Two different sequence sets were used for depicting the phylogenetic relationships between the Vo’ samples and the European epidemiological framework. The first sample set included a total of 1252 full genome sequences from Europe, collected in the same pandemic period. The second data set included a selection of sequences from the first set (*n* = 148). Both sets included SARS-CoV-2 Wuhan-Hu-1 isolate (Genbank accession number MN90894) as a reference (GenBank accession number: MN908947.3). The 90 bp end at both 5′ and 3′ were eliminated to avoid possible sequencing errors. Phylogenetic trees were constructed using RAxML [16] software. Each tree is statistically supported by the bootstrap process (100 replicates) and the Wuhan isolate was used as the outgroup.

### 2.6. Metadata Collection

At the surveys, subjects were interrogated about the type and timing of symptoms. Moreover, contact tracing information was collected according to the general Centers for Disease Control and Prevention (CDC) guidelines [17]. The subjects were asked for information on the duration and type of contact and the places visited at that time. Full household information was further provided by the town hall.

## 3. Results

### 3.1. Viral Haplotypes Circulating in Vo’

During the first surveys, we collected nasopharyngeal swab samples from nearly the entire population of Vo’ at two consecutive time points, (21–29 February 2020 and 7 March 2020). We sequenced the viral genomes from 75 subjects, with 12 of them testing positive at both timepoints, for a total of 87 sequences, which were uploaded to GISAID [18].

**Figure 1:**
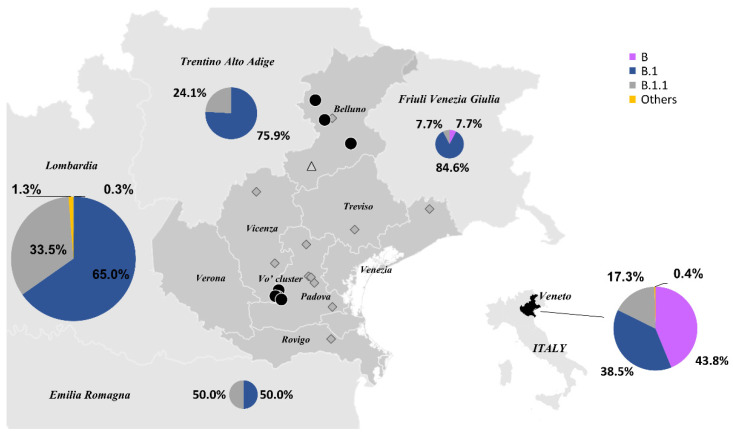
Lineages circulating in the northern Italian regions. The percentages of SARS-CoV-2 lineages circulating in the Emilia Romagna (*n* = 2), Friuli Venezia Giulia (*n* = 13), Lombardia (*n* = 400), Trentino Alto Adige (*n* = 58), and Veneto (*n* = 226) regions in February and March 2020, according to GISAID database. The sizes of the pie charts reflect the number of sequences available per region. The different lineages are colored according to the legend. Black circles represent haplotypes identical to those found in Vo’, diamonds indicate haplotypes with one additional mutation (edit distance +1 from Vo’), and triangles correspond to haplotypes with two additional mutations (edit distance +2 from Vo’).

All the viral sequences were characterized by two mutations, G11083T and G26144T (from here on reported as the ancestor haplotype, AH). Interestingly, these mutations were previously identified in a couple of Chinese tourists who, after visiting Verona, Parma, and Florence, were diagnosed with SARS-CoV-2 infection in Rome on 31 January 2020 and correspond to the first reported cases of COVID-19 in Italy [19]. Surprisingly, this haplotype, classified as lineage B according to Rambaut’s nomenclature [20], accounted for 43.8% of the total sequences from February to March 2020 in the Veneto region. In the surrounding regions, such as Lombardia and Trentino Alto Adige [21], we found evidence of a different source of introduction, given that the B.1 lineage was dominant [22] (Figure 1).

While the AH was found only in Vo’ and in the province of Belluno, some of AH’s derivative subtypes were observed in other provinces of the Veneto region.

The sequencing data indicate that the B lineage haplotypes introduced in the Veneto region, like the ones identified in Vo’, derive from the AH, carrying G11083T and G26144T mutations that further developed new additional mutations.

According to the sequenced data, the AH was prevalent in Vo’ (38 out of 87, 43.7%), while an additional 26 unique haplotypes derived from the ancestral one. Of the 42 unique point mutations defining the different haplotypes (including G11083T and G26144T) mapped along different regions of the viral sequence, 1 was in the 5′-UTR (2.4%), 15 were synonymous (35.7%), and 26 were non-synonymous (61.9%). A 9 nucleotide deletion was observed in 2 samples, resulting in a 3 amino acid deletion affecting non-structural protein 1 (nsp1) (ORF1a:K141-,ORF1a:S142-,ORF1a:F143-). The deletion of these amino acids was suggested to affect viral replication and host gene expression, and was further observed in other sequenced SARS-CoV-2 genomes belonging to different lineages [23] and different countries, implying homoplasy events. 

Although the short time window of observation hampered the detection of a natural or purifying selection, most of the non-synonymous mutations found in Vo’ emerged independently later during the pandemic in different countries (Table S1). The two mutations defining the AH behaved differently during the pandemic. G26144T was mostly associated with G11083T in lineage B and disappeared in April 2021 after reaching a peak at the beginning of the pandemic (February–April 2020). Conversely, the G11083T mutation appears in recently collected sequences belonging to different lineages, suggesting that this is another example of homoplasy [24] (Figure S1).

Interestingly, the mutation Spike:L5F (C21575T), which characterized the viral haplotype of two subjects in Vo’, emerged from January 2021 to August 2021 as part of the mutation pattern defining the B.1.526 lineage (Iota variant), prevalent in the USA [25] (Figure S2).

At the gene level, G26144T mutation leads to an NS3:G251V amino acid change, which was reported to increase disease severity by 4.4 times [26]. G11083T (NSP6:L37F), on the other hand, has been hypothesized to prevent the fusion of the autophagosomes to the cell lysosomes, affecting viral replication and evasion from cellular immunity [27,28]. Studies on Spike:L5F highlighted the increase in the protein folding and assembly of virions [29], as well as the increase in CD8 T cell recognition and killing [30].

### 3.2. Vo’ Haplotypes in Europe

We compared the viral genomes collected in Vo’ with a selection of contemporary European sequences from different locations. At the beginning of the pandemic, the AH was also circulating in other European countries, with the UK, Italy (90% in the Veneto region), and Spain reporting the highest occurrences. To analyze the data, we used two different approaches based on phylogenetic analysis (maximum likelihood, ML, phylogenetic tree) and on the construction of a minimum spanning network, MSN (see Methods). 

**Figure 2:**
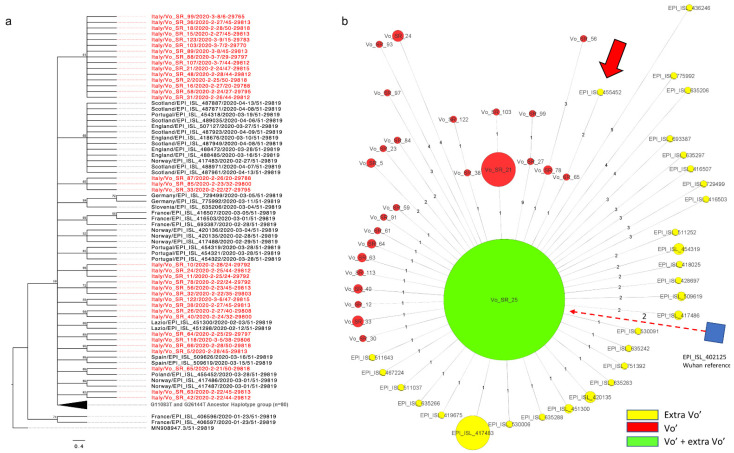
Maximum likelihood (ML) phylogenetic tree and minimum spanning network (MSN) of sequences related to Vo’ viral genomes. The European sequences closely related to the Vo’ viral genomes and with collection dates corresponding to the beginning of the pandemic were retrieved from GISAID and utilized for the ML phylogenetic tree and MSN. (**a**) Maximum likelihood phylogenetic tree. The phylogenetic tree is collapsed in correspondence of the ancestor haplotype (AH), which was found both in Vo’ and other European countries. The sequences from Vo’ are reported in red whereas the black ones are from Europe. (**b**) Minimum spanning network. The AH is represented as a big green node containing both the Vo’ and European sequences. The sizes of the nodes reflect the abundance of identical sequences. The distances of the edges from the central node reflect the number of accumulated mutations. The European/Italian sequences are reported in yellow, the Vo’ sequences are reported in red, and the Wuhan reference node is represented as a blue square. The only extra-Vo’ haplotype genetically related to a Vo’ haplotype is indicated by the thick red arrow and the dashed arrow links the Wuhan reference genome to the AH.

In Figure 2, we provide an ML phylogenetic tree and an MSN limited to Vo’ haplotypes and to closely related European haplotypes, where all the European sequences carrying the AH, including the Italian ones, are represented as a big green node in the MSN, while they are collapsed in the phylogenetic tree. The same analysis, extended to consider all the European sequences collected at the time, are provided in Figures S3 and S4 for the phylogenetic tree and the MSN, respectively. According to the phylogenetic and MSN reconstructions, only one sequence, “VO_SR_65” (haplotype G11083T, G26144T, C22088T), shares a private mutation with a Polish sequence, EPI_ISL_455452 (haplotypes C4338T, T9743C, G11083T, G26144T, and C22088T). However, the Polish sequence (dated 2020-03-28) relates to a sample collected one month later than the Vo’ sample “VO_SR_65” (dated 21 February 2020), suggesting an independent evolution and possibly a homoplasy event.

These results confirmed that the Vo’ haplotypes were not observed in other countries and did not generate descendants, thanks to the mass testing and lockdown strategy implemented in Vo’ [31,32,33].

### 3.3. Intra-Host Viral Evolution

**Figure 3:**
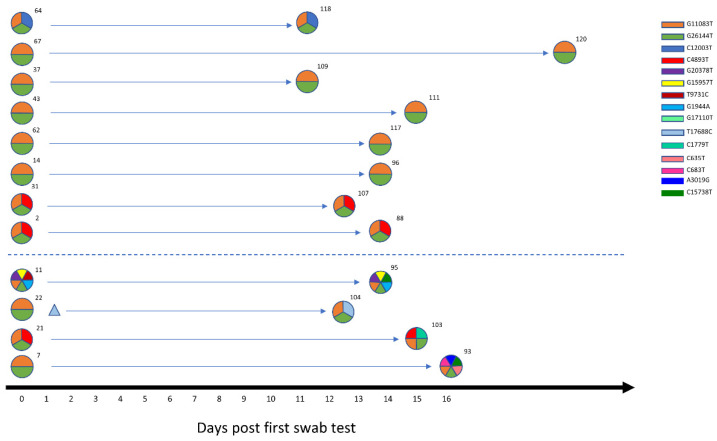
Within-host variation. A longitudinal analysis of SARS-CoV-2 intra-host variation in 12 subjects in an average time window of 11 days. The viral haplotypes of the two consecutive swab tests of each subject are reported on the same line. Each haplotype is depicted as a circle, with each slice representing a mutation, characterized by a color according to the legend. Minor variants are represented as triangles. The dotted line separates evolved haplotypes from the stable haplotypes.

We obtained the viral sequence of 12 individuals who tested positive at two sequential swab tests. As reported in Figure 3, in 4 out of 12 subjects (33.3%) the virus acquired at least one novel mutation in an average time of 11 days (range 9–16 days). While 3 out of 4 individuals acquired a single mutation at the second time point, 1 subject (samples VO_SR_7 and VO_SR_93) accumulated 4 mutations in 13 days, highlighting the rapidity of viral evolution in some subjects. No significant differences in days after the first swab test, disease severity, antibody production, or T cell receptor breadth or depth [6] were found when comparing individuals with a constant viral haplotype to individuals with a mutated viral haplotype (Figures S5 and S6). Given that all the haplotypes identified in Vo’ derive from the AH and that within approximately two weeks of positivity the viral genomes accumulated at least 1 novel mutation in 33.3% of cases, we further investigated this event among all the available subjects. Considering that in Vo’ we identified 26 unique haplotypes (AH excluded) out of 87 subjects and the two week lockdown imposed by the local authorities, we can assume that intra-host evolutionary events occurred in 26 subjects out of 87 (29.9%) within this time window. Of these 26 cases, 4 were proven by the sequential swab tests, as described above, while another 10 cases could be inferred according to the viral haplotype evolution within family households, virus haplotypes, and the symptom onset dates (Table 1).

### 3.4. Within-Household Variability

The different haplotypes identified in Vo’ were analyzed in the context of the household structure to investigate the potential transmission chains and within household viral evolution.

**Figure 4:**
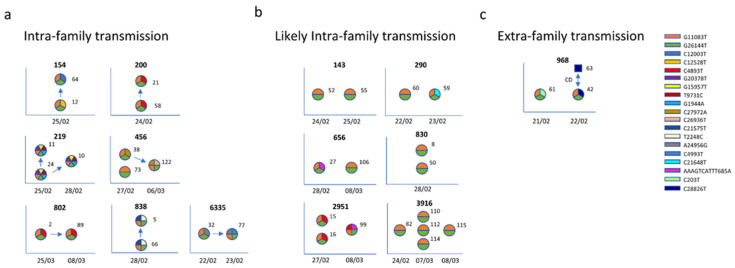
Within-family diversity and transmission. The sequences were grouped according to the households and analyzed separately. Each haplotype is depicted as a circle, with each slice representing a mutation, characterized by a color according to the legend. For each household (identification number provided at the top of each diagram) the genetic information of family members (labeled with an identification number) and relative metadata (date of positive swab tests reported on the *x* axis) were utilized to investigate the diversity and the transmission chain. Households were then subdivided into within-household transmission (**panel a**), uncertain transmission (**panel b**), and extra-household transmission, where the direct contact (CD) is represented as a blue square (**panel c**).

The intra-family transmission chains reported in Figure 4, and extensively explained in Appendix A, were based on: (a) swab test results and relative dates, (b) viral consensus genome, and (c) minor variants, considered as alternative nucleotides with a frequency from 5% to 49%. We assumed that the subjects who negativized earlier were the infectors and assumed no reversion.

Interestingly, we found evidence that a few mutations were household-specific (not present in any other sample collected in Vo’ outside the house). Households 838 and 219, where 2 out of 2 and 3 out of 3 family members shared the same haplotypes (G11083T, G26144T, T2248C, and C21575T and G11083T, G26144T, G1944A, T9731C, G15957T, and G20378T, respectively), exemplify this phenomenon.

Household 456, where one household member was characterized by the AH (haplotype G11083T and G26144T), the second one acquired a novel private mutation (haplotype G11083T, G26144T, and C27972A), and the third one further acquired another private mutation (haplotype G11083T, G26144T, C27972A, and C26936T), likely reflects the direction of transmission and the sequential viral evolution during transmission among household members. 

By enriching the genetic data with metadata, such as swab tests and contact information, it was possible, for 8 out of 14 (57.1%) households, to reconstruct the household transmission chains, which are depicted with arrows in Figure 4 panels ‘a’ and ‘b’. Notably, while seven out of eight of the reconstructed transmissions occurred within the same household (Figure 4a), in one case we found evidence of household members contracting the infection outside of the household. This was confirmed by the difference in viral haplotypes between the two household members, with one of them sharing the same haplotype with a declared direct contact (Figure 4c). In the remaining six households, although the transmission probably occurred within family members, we did not have enough information to reconstruct the transmission chains (Figure 4b). 

### 3.5. Transmission Chain Reconstruction

**Figure 5:**
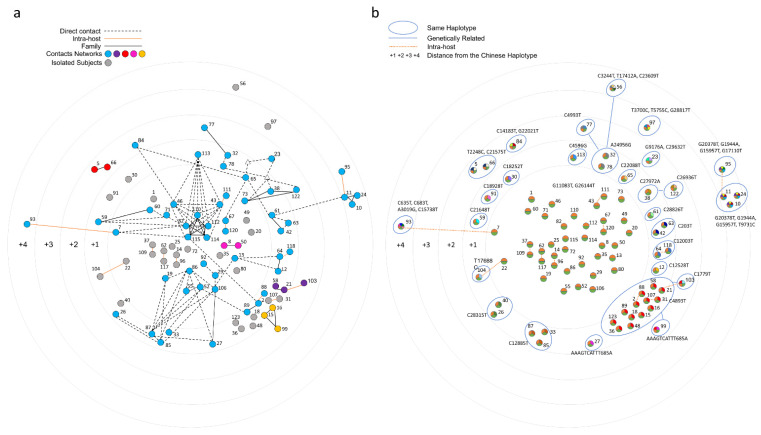
Network of contacts based on post-infection interviews. (**a**) Samples are connected according to the contacts declared during the post-infection interviews. Each contact chain is colored according to the legend, with nodes not declaring any contact being colored in grey. Subjects who declared informative contacts, but without an available viral sequence, are depicted as white triangles. (**b**) Vo’ haplotypes are clustered according to their mutations and the genetic distance from the Ancestor Haplotype (edges at distance 1 are not drawn for graphical reasons).

In Figure 5, contact (panel on the left) and genetic (panel on the right) data are reported and compared. 

Given the incompleteness of the contact information and of the reported dates of symptom onset, it was not possible to unequivocally identify the chains of transmission events in Vo’, even with the support of genetics. However, the data suggest that the outbreak initiated from an indoor space that fueled a superspreading event. The time-lapse animation (see Movie S7) shows the reported contacts according to the reported times of symptom onset. 

## 4. Discussion

The population of Vo’ was the first in Italy and Europe to be placed under lockdown by the regional authorities after the first ascertained COVID-19 death in the country, providing the opportunity to study in detail different aspects of the epidemic in a controlled epidemiological setting.

The sequencing data identified the Vo’ Ancestor Haplotype (AH), characterized by the G11083T and G26144T mutations, from which all the other sequenced haplotypes evolved. This haplotype, initially observed in two Chinese tourists visiting Italy in January 2020, was dominant in the Veneto region at the beginning of the pandemic, while lineage B.1 was prevalent in the surrounding regions, revealing multiple viral introductions in Italy [34]. 

Moreover, MSN and phylogenetic analyses including European sequences from the same period confirmed that, although the AH was also circulating in other countries, none of the descendant haplotypes evolved in Vo’ were observed elsewhere. Considering the absence of vaccines or treatments at that time and the uncertainty related to the virus and its relative disease, the lockdown was efficient in suppressing the outbreak in Vo’ and the new virus variants that emerged from it. Interestingly, we observed three cases of putative homoplasy, with: (a) C22088T, present in a Polish sequence collected one month later than Vo’ outbreak, (b) G11083T, found in several sequences from different countries and lineages, and (c) C21575T, shared by two Vo household members and appearing at a global level only in 2021, as a mutation defining the B.1.526 lineage. 

According to the present literature, the accumulation of viral mutations at the consensus level was previously observed in treated [35,36] or immunocompromised subjects [37,38] experiencing prolonged infections, while other studies reported only longitudinal changes of intra-host minor variants [39,40]. Here, we provide the first evidence of the rapid intra-host accumulation of mutations at the consensus level in a time window as short as two weeks, regardless of symptom severity or immunodeficiencies. The generation of new haplotypes occurred in an average time of 11 days in 33% of the samples (4 out of 12) in otherwise healthy individuals. 

In an exemplary case, a total of 4 mutations accumulated in 13 days, pointing out the rapidity of viral evolution. Based on the collected data, we could infer a frequency of intra-host evolution of 30% (26 out of 87). Although no correlation with symptom severity, length of the infection, or serological data was found, other mechanisms could be responsible for within-host viral evolution, such as the host immune response [41,42].

Given the frequency of intra-host viral evolution, it was difficult to define a transmission chain based on genetics, since the differences in the haplotypes can derive either from the haplotype carried by the infector or by an intra-host evolution that occurred in the infected individual. The integration of sequencing data with metadata allowed us to infer the transmission chains among some household members, indicating within household transmissions as the most frequent transmissions (7 out of 14).

When attempting to reconstruct a transmission chain, we faced the drawbacks of contact tracing based on interviews. Undoubtedly, the declared contacts were incomplete, due to both psychological factors, such as stress, anxiety, or embarrassment, and to the limits of recalling from memory [43]. Moreover, the personal perception of events and contacts led to an overreporting of symptomatic positive friends and an underreporting of household members. Consequently, we decided to complement this data with information provided by the town hall and with symptom data.

Though the contact tracing analysis was limited to symptom onset data to reconstruct a temporal expanding network of infected subjects, it enabled us to identify the first infections and a gathering place that had a key role in the transmission of the infection.

To sum up, this study sheds light on the effectiveness of contact tracing based on interviews, which showed limitations in the coverage and traceability of the contacts among the identified infections. For these reasons, in the absence of effective digital contact tracing, mass testing followed by case isolation represents the best approach, when applicable, for identifying and isolating infections, thus giving the best chances to achieve epidemic control [44,45,46]. As a matter of fact, the variants generated in the Vo’ population did not spread outside Vo’, showing that effective control strategies not only curb transmission, but also control the emergence and spread of new variants. The number of mutations observed in the Vo’ population over just two weeks should serve as a warning about the velocity of adaptation that may be occurring in different subjects and the potential ability of SARS-CoV-2 to develop immune evasion.

## Figures and Tables

**Table 1:** Inferred intra-host viral evolution.

	SUBJECTS	NOTES
**HOUSEHOLDS**	SR_38, SR_122, SR_64, SR_12, and SR_77	Subjects infected by family members carrying a different haplotype
**HAPLOTYPES**	SR_56 and SR_99	Subjects carrying a haplotype that evolved from a subtype of the AH
**SYMPTOM ONSET DATES**	SR_65, SR_61, and SR_30	First subjects contracting the infection, likely infected with the AH

## Data Availability

The data presented in this study are openly available in a github repository at the following link: https://github.com/MedCompUnipd/SARS-CoV-2_Vo_genomics.git (accessed on 13 January 2022).

## Supplementary Materials

The following supporting information can be downloaded at: https://github.com/MedCompUnipd/SARS-CoV-2_Vo_genomics.git, Figure S1: Mutations defining the Vo’ haplotype in time; Figure S2: S:L5F appeared in Iota variant in 2021; Figure S3: ML phylogenetic tree of the European lineage B SARS-CoV-2 sequences at the beginning of the pandemic; Figure S4: Minimum spanning network of the European lineage B SARS-CoV-2 sequences at the beginning of the pandemic; Figure S5: Antibody titers of subjects positive to two sequential swab tests with an average time interval of 11 days; Figure S6: TCR features of subjects positive to two sequential swab tests with an average time interval of 11 days; Table S1: Vo’ non-synonymous mutations according to different clades; Table S2: Correspondence of GISAID IDs, patients’ symptoms with their corresponding viral haplotypes, and mutations found in the sequenced viral genomes; Video S1: Expanding network based on the symptom onset data; GISAID acknowledgement table.

## Appendix A. Intra-Family Transmission, a Detailed Description

Among the 14 households sequenced, a singular case distinguished household 154, where the two family members (12 and 64) had different private mutations (C12528T and C12003T, respectively). By investigating the minor variants, the private mutation appearing in the consensus genome of subject 64 (C12003T) was found at a frequency of 19% in subject 12. According to the results of swab testing, subject 12 negativized earlier than subject 64 (2 March versus 9 March), suggesting that subject 12 was the first one to contract the infection, harbored both private mutations (C12528T and C12003T), and infected subject 64 (when none of these private mutations were present at the consensus level), with C12528T affirming in subject 12 as the major variant and C12003T affirming as the major variant in subject 64. This highlights the host selection of viral mutations and suggests the co-existence of quasispecies with one prevailing over the others due to host selection.

A simpler picture describes households 200 and 802, where all the family members shared the same viral haplotype (G11083T, G26144T, and C4893T), and the infection chain was determined by the swab test results. Specifically, subject 58 negativized earlier than subject 21 (first negative swab test 2 of March), with subject 21 still testing positive on 7 of March, suggesting that subject 58 infected subject 21.

In household 802, subject 89 was negative on 26 of February, while subject 2 was positive the day before (25 February). Thus, we inferred that subject 2 infected subject 89.

Subject 24 of household 219 was the first one to have a negative swab test (7 March) and shared the same viral haplotype as the other family members (samples 10 and 11, haplotype G11083T, G26144T, G1944A, T9731C, G15957T, and G20378T), who were still positive on 9 March and were, thus, probably infected by subject 24.

As described in the main text for household 456, viral evolution is further supported by the chains of transmission identified from the swab test results, with subject 122 (haplotype G11083T, G26144T, C27972A, and C26936T) being negative on 27 February, when subjects 38 (haplotype G11083T, G26144T, and C27972A) and 73 (haplotype G11083T and G26144T) were already positive. Due to the private mutation shared by subjects 122 and 38, it is reasonable to conclude that subject 38 infected subject 122. Considering that subject 73 showed the private mutation C27972A as a minor variant (frequency 6%), we could not determine the index case in the household between subject 38 and subject 73.

Regarding household 838, subject 66 negativized on 7 March, and we inferred that they infected subject 5, who tested positive on 9 March.

For household 6335, while both subjects tested positive at the same time (22 and 23 February), the viral haplotypes indicated that subject 32 (haplotype G11083T, G26144T, and A24956G) infected subject 77 (haplotype G11083T, G26144T, A24956G, and C4993T).
