# Supplementary material for: Rapid SARS-CoV-2 Intra-Host and Within-Household Emergence of Novel Haplotypes
Source: Viruses. 2022 Feb 15;14(2):399. doi: 10.3390/v14020399 (PMC8877413; doi:10.3390/v14020399)
Supplement: Supplementary file 1 [file viruses-14-00399-s001.zip › SupplementaryMaterial.pdf]

viruses

Supplementary materials

Rapid longitudinal SARS-CoV-2 intra-host emergence of novel haplotypes regardless of immune deficiencies

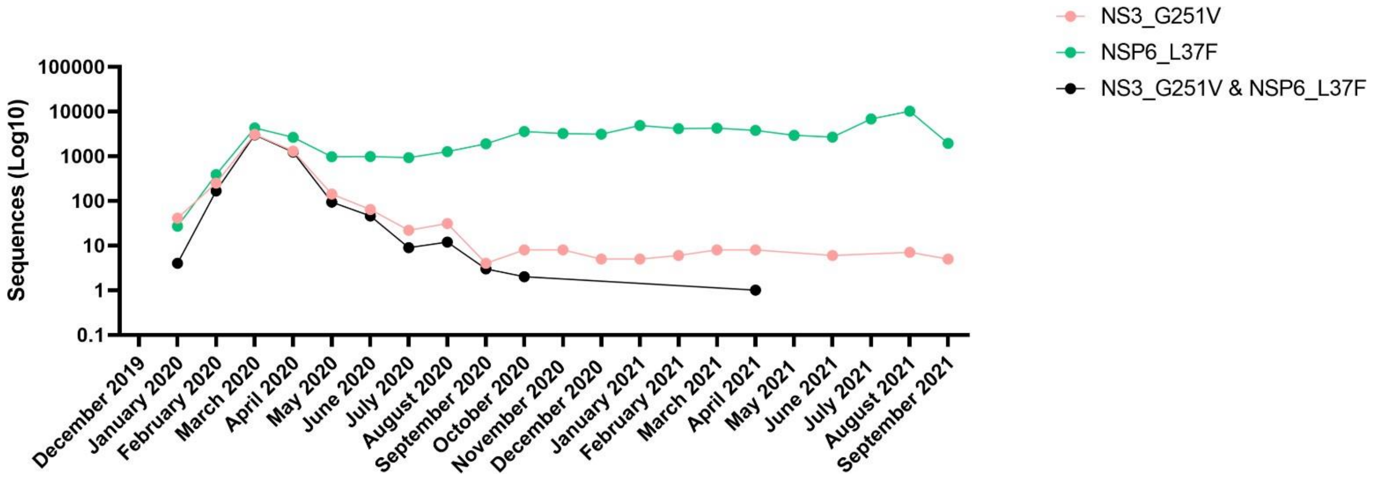

Figure S1. Mutations defining Vo' haplotype in time. Number of sequences carrying G11083T (NSP6:L37F) and G26144T (NS3:G251V) mutations deposited in the GISAID database from December 2019 to September 2021. Mutations were analysed separately (green and pink line) and as part of the same haplotype (black line).

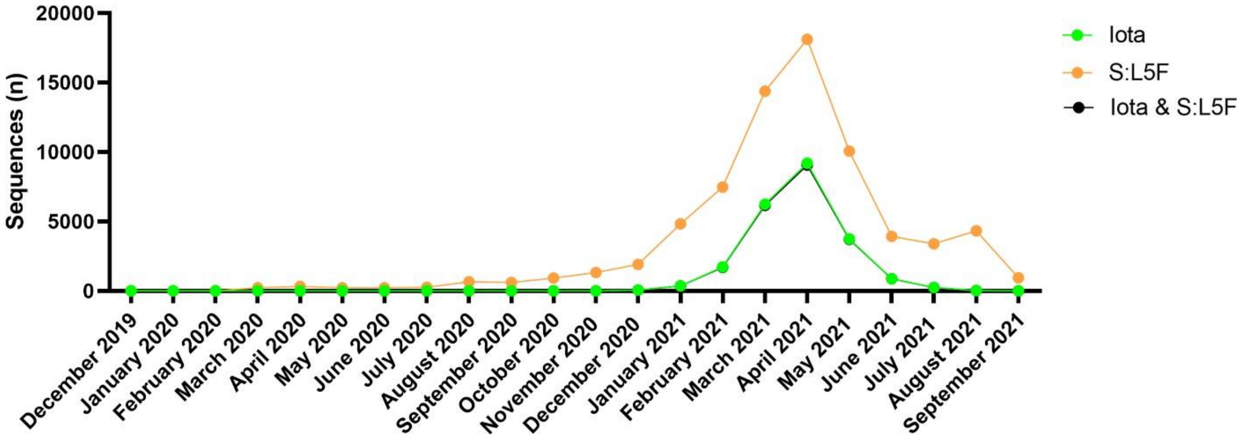

Figure S2. S:L5F appeared in Iota variant in 2021. Number of sequences deposited in the GISAID database and collected from December 2019 to September 2021 carrying C21575T (S:L5F) mutation (yellow line) compared to sequences belonging to 21F clade (Iota variant, green line) and the combination of the two cases (black line).

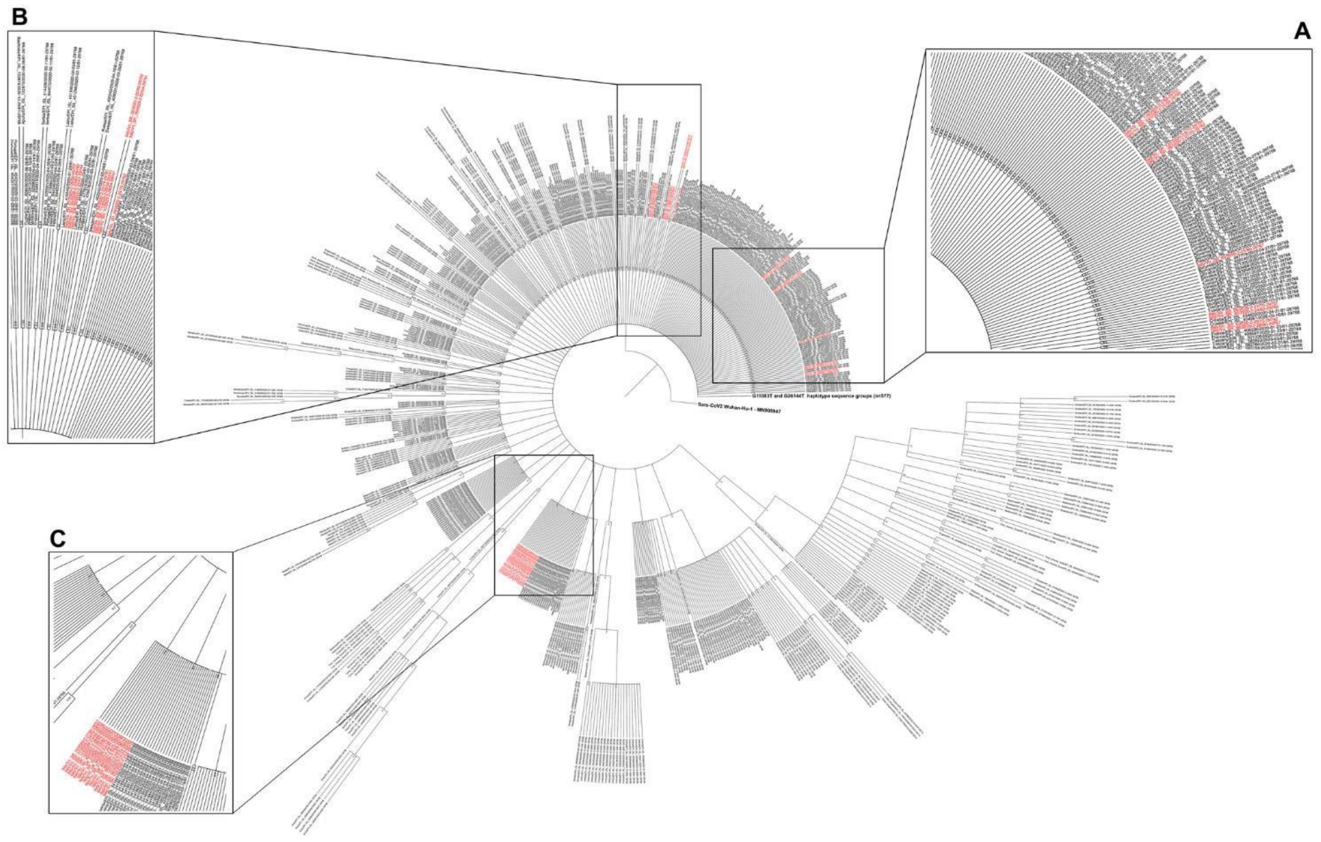

Figure S3. ML phylogenetic tree of European lineage B SARS-CoV-2 sequences at the beginning of the pandemic. Maximum likelihood phylogenetic tree of all European sequences collected at the beginning of the pandemic and uploaded in GISAID. Vo' sequences are coloured in red. All sequences carrying the Ancestor Haplotype are collapsed. A, B and C panels zoom in the subtypes of the ancestor haplotype observed in Vo'.

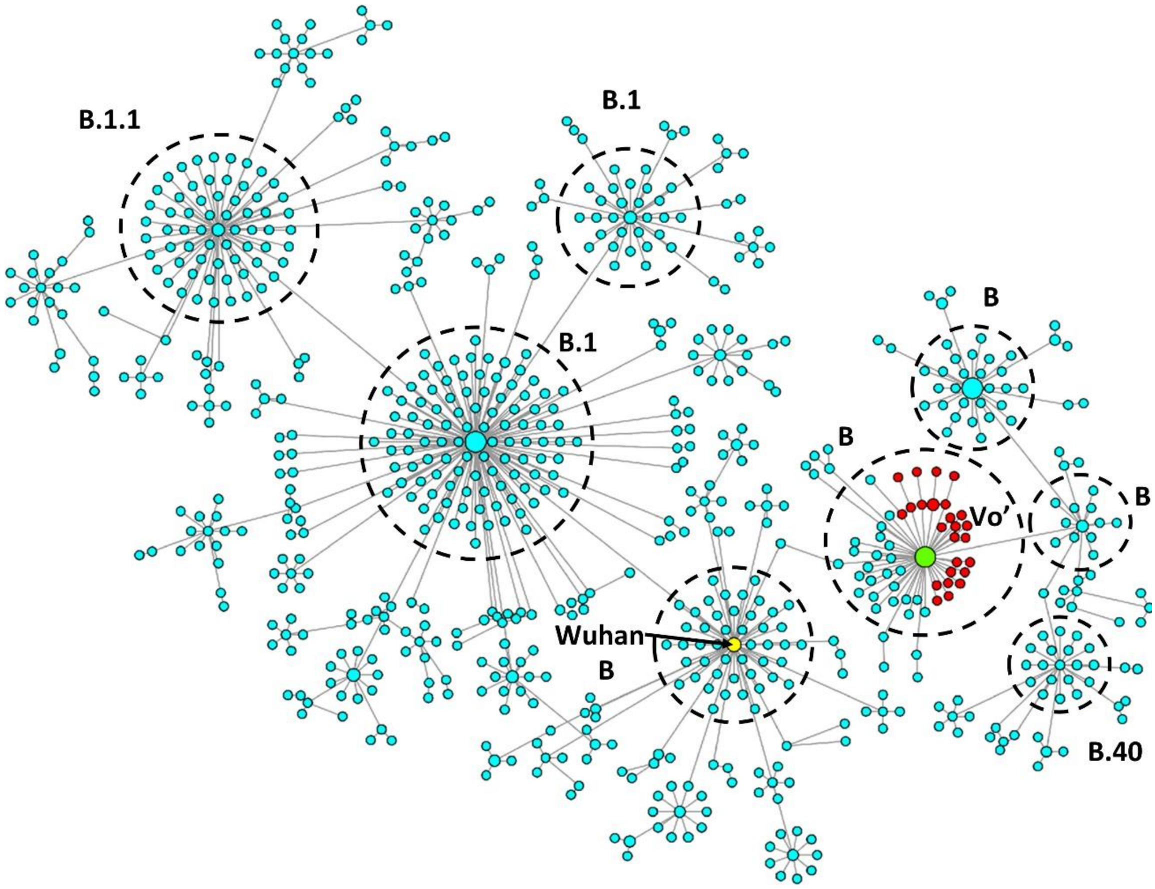

Figure S4. Minimum Spanning Network of European lineage B SARS-CoV-2 sequences at the beginning of the pandemic. MSN of all the European sequences collected at the beginning of the pandemic and uploaded in GISAID. Each dot represents a unique haplotype, with Vo' haplotypes coloured in red. Haplotypes are clustered according to the lineage they belong to, with each cluster being circled with a black dotted line, and showing the basic haplotype in the central node.

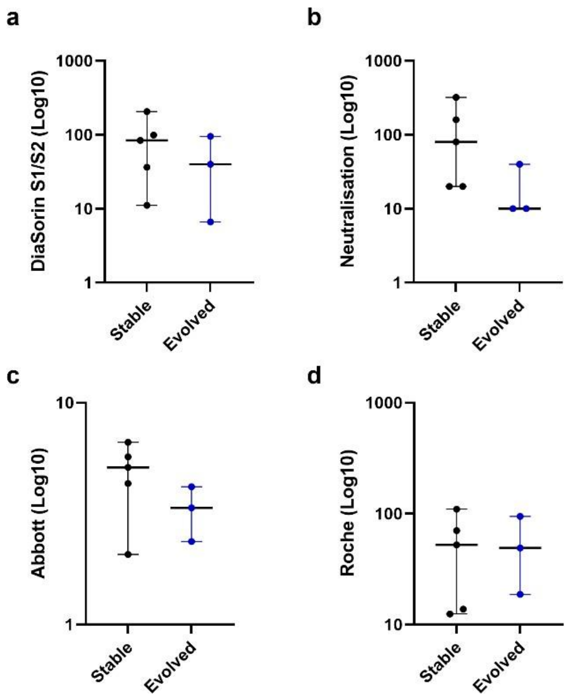

Figure S5. Antibody titres of subjects positive to two sequential swab tests with an average time interval of 11 days. Antibody titres observed in subjects with stable (black) and unstable (blue) viral genome in an average time span of 11 days according to DiaSorin, Neutralisation, Abbott and Roche assays (Mann Whitney test).

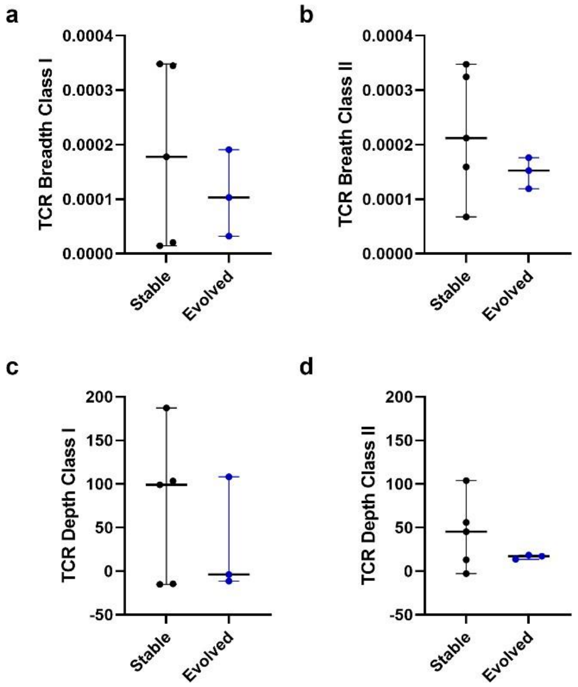

Figure S6. TCR features of subjects positive to two sequential swab tests with an average time interval of 11 days. TCR breadth class I (a) and class II (b) of patients with stable (black) and unstable (blue) viral genome in an average time span of 11 days (Mann Whitney test). TCR depth class I (c) and class II (d) of patients with stable (black) and unstable (blue) viral genome in an average time span of 11 days (Mann Whitney test).

https://github.com/MedCompUnipd/SARS-CoV-2_Vo_genomics/blob/c3031676e62178da544d9aec88b63393ba875e5f/Movie_S1.mp4

Movie S1. Expanding Network based on symptoms onset data. Movie based on symptoms onset data reported by the infected individuals regardless of the availability of the viral sequences. Subjects reporting contacts are depicted as rectangles, while circles represent individuals reporting no contacts with the subjects traced in the main chain. Nodes with an available sequence are coloured in green, otherwise they appear in red. Individuals who did not report a symptom onset date appear in the last frame all together.
